# Shoot-branching number control: insight for soybean architecture breeding

**DOI:** 10.3389/fpls.2026.1987498

**Published:** 2026-10-23

**Authors:** Meng Li, Xiao Chen, Yawar Habib, Javaid Akhter Bhat, Elena Potokina, Xianzhong Feng

**Affiliations:** 1 Sanjiang Lab, Jilin Agricultural University, Changchun, China; 2 Key Laboratory of Soybean Molecular Design Breeding, Northeast Institute of Geography and Agroecology, Chinese Academy of Sciences, Changchun, China; 3 Center for Bio and Medical Technologies, Skolkovo Institute of Science and Technology, Moscow, Russia; 4 College of Advanced Agricultural Sciences, University of Chinese Academy of Sciences, Beijing, China

**Keywords:** gene regulation, molecular design breeding, QTLs, shoot branching number, soybean (*Glycine max*)

## Abstract

Shoot branching in plants (including tillering in graminaceous crops) is a key agronomic trait affecting plant architecture, resource allocation, and final yield. Branching is a complex developmental process modulated by genetic networks regulating axillary meristem (AM) initiation and subsequent bud activation. This review summarizes the genetic regulatory networks identified in model plants, specifically *Arabidopsis thaliana* (a dicot) and *Oryza sativa* (a monocot). Building on this cross-species theoretical framework, we focus on soybean (*Glycine max*), a major economic crop, and provide a comprehensive analysis of recent progress in the functional characterization of branch number specific regulators and quantitative trait loci (QTLs). Given the bottlenecks in soybean architecture improvement resulting from its complex and highly redundant genome, recalcitrance to genetic transformation, and high environmental sensitivity, we discuss potential strategies based on multidisciplinary approaches and emerging technologies. This review aims to provide a new insight for shaping the ideal plant architecture in soybean through molecular design breeding.

## 1 Introduction

The branching of plants’ shoots (also referred to as tillering in graminaceous crops) is a developmental process involving the initiation and elongation of lateral buds. This trait is a key determinant of plant architecture as well as of final crop yield (Barthélémy and Caraglio, 2007; Teichmann and Muhr, 2015; Clark and Ma, 2023; Hou et al., 2026). The branching has been revealed to plays a vital role in shaping plant morphology, regulating resource allocation, and facilitating adaptation to complex environmental changes (Freschet et al., 2018; Gu et al., 2026; Hu et al., 2026). The optimum branching pattern is important for increased photosynthesis, which in turn leads to higher biomass and overall productivity (Soualiou et al., 2021; Liu et al., 2024; Cai et al., 2026). Moreover, under fluctuating environmental conditions, plants modulate their branching pattern for optimal distribution of resources, resulting in more efficient nutrient uptake and improved resilience against abiotic stresses (Singh et al., 2024). In agriculture, the breeding objective for branch number varies among different crop species; for example, in some crops the strategy is to increase branch number for yield maximization, whereas in other crops a reduced branch number is preferred for compact plant architectures and improved population-level economic returns (Li W. et al., 2024). Hence, it is a prerequisite to elucidate the detailed molecular mechanisms underlying plant branching to precisely modulate this trait according to the breeding objectives.

The development of plant branches is a complex and highly regulated process that involves two key steps (**Figure 1**): (a) the initiation and formation of axillary meristems (AM) at leaf axils, and (b) the development of AM into axillary buds, which may either undergo outgrowth or remain dormant (Domagalska and Leyser, 2011). This process is controlled by an extensive and sophisticated regulatory network, in which genetic factors play a central role. In recent years, studies conducted in model plants such as Arabidopsis (*Arabidopsis thaliana*) and rice (*Oryza sativa*) have identified many core genes regulating AM initiation and bud elongation, including *LATERAL SUPPRESSOR* (*LAS*), *MONOCULM 1*(*MOC1*), and *BRANCHED1* (*BRC1*) (Wang et al., 2018). Besides, it has been revealed that different plant hormones (viz., auxin, cytokinin, and strigolactones), environmental factors and nutritional signals interact with these genes to determine branch development (Mason et al., 2014). The regulatory basis of branch number identified in these model plant species can be used for the translational research of non-model crop species such as soybean.

**Figure 1:**
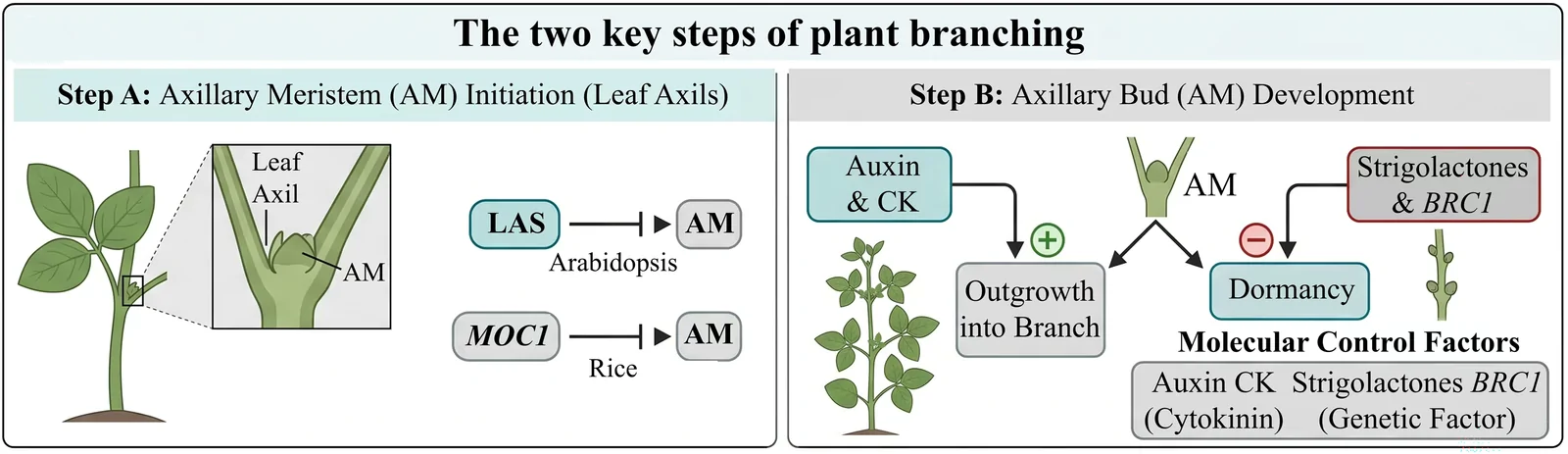
Schematic representation of the morphology and key developmental stages of plant branching.

Soybean (*Glycine max*), an important source of edible oil and protein, possesses a plant architecture in which branch number directly determines canopy closure, light interception, and ultimately, effective pod number and yield. The heritability of plant architectural traits including branch number, branch length and branch angle determines the efficiency of phenotypic selection in soybean breeding. Generally, soybean branch number and branch angle exhibit moderate to high heritability, indicating that a large proportion of their phenotypic variation is genetically controlled and these traits can be readily improved via selection (Gao et al., 2017; Sun et al., 2023; Cai et al., 2026; Gu et al., 2026). By contrast, the heritability of branch length is still unclear and it might be more susceptible to environmental fluctuations such as planting density and soil fertility. Heritability estimates vary across populations, environments and developmental stages, because genotype-by-environment (G×E) interactions frequently shape the expression of branching-related traits (Yu et al., 2024; Gu et al., 2026). In comparison to the model plants such as rice and Arabidopsis, where the molecular regulatory mechanism of shoot branching has been well elucidated, efforts made in the soybean for the elucidation of molecular mechanisms in oilseed crops like soybean remain relatively limited (Clark and Ma, 2023; Hou et al., 2026). Manipulating branch number to develop the “ideal plant architecture” in soybean suitable for high-density planting therefore remains a significant scientific challenge.

In this context, the aim of the current review is to systematically synthesize the molecular regulatory mechanisms of plant branching. We first discuss the core gene networks and hormonal interplay governing AM initiation and bud outgrowth in Arabidopsis and rice. Considering the conservation of gene function across plant evolution, we then discuss how these core genes can inform soybean improvement. Accordingly, we focus on soybean and comprehensively analyze current research progress on branching-related genes and quantitative trait loci (QTLs). We further explore the potential mechanisms of multi-factor regulatory networks in soybean branching, and provide an in-depth analysis of the challenges and future directions for soybean architecture improvement.

## 2 Molecular regulatory mechanisms of branching in Arabidopsis

The model plant *Arabidopsis thaliana*, widely used for research on dicotyledonous plants, has undergone substantial research aimed at elucidating the genetic basis of branch number. Hence, this plant species has accumulated the most comprehensive data regarding the genetic basis of branching development. In Arabidopsis the development of lateral branches is strictly divided into two successive biological processes: (a) the *de novo* initiation of axillary meristems (AM) at the leaf axils, and (b) the subsequent activation and elongation of dormant axillary buds regulated by internal and external signals.

### 2.1 Genetic networks governing axillary meristem initiation

The formation of AM is the basis of plant branching, and this process relies on the specification of boundary cells at the leaf axil junction and the establishment of a *de novo* stem cell niche (Wang et al., 2014; Nicolas and Laufs, 2022a). In Arabidopsis, this process is coordinately regulated by multiple transcription factors belonging to different families via a rigorous spatiotemporal cascade. For AM initiation, there is a prerequisite necessity for the precise and well-defined formation of the boundary zone, which is primarily orchestrated by the transcription factors belonging to the NAC family viz., CUC2 and CUC3 (Li et al., 2020). For example, it has been well documented that the double mutant viz., *cuc2cuc3* shows the complete abolition of AM initiation, resulting in the absence of lateral branches (Hibara et al., 2006; Aslam et al., 2022). Although, the *CUC* genes are essential for the establishment of the boundary zone between leaf primordia and the primary shoot axis, their continuous expression hinders the reprogramming and activation of pluripotent stem cells (Hibara et al., 2006). In this regard, as development progresses, the NGATHA-LIKE transcription factors viz., DPA4/NGAL3 and SOD7/NGAL2 redundantly repress *CUC* expression in initiating AMs of *Arabidopsis thaliana*; SOD7 has been shown to directly bind the CUC3 promoter, whereas repression of CUC2 involves NGAL-mediated regulation (Nicolas et al., 2022; Nicolas and Laufs, 2022b).The absence of NGATHA-like-mediated repression leads to ectopic expression of *CUC2/3*, which results in a severe delay in the transcription of core meristematic genes viz., *WUSCHEL* (*WUS*) and *CLAVATA3* (*CLV3*) (Nicolas et al., 2022); these circumstances are causing the obstruction of *de novo* stem cell establishment and resulting defective axillary bud initiation or a significantly reduced branching phenotype (Nicolas et al., 2022). In contrast, the early activation of *CUC* genes, followed by their precise inhibition by the DPA4/SOD7 transcription factors, constitutes a critical regulatory switch that enables the transition from organ boundary identity to *de novo* stem cell establishment, a critical genetic switch for the transition from “boundary specification” to “stem cell reprogramming” (Nicolas et al., 2022; Nicolas and Laufs, 2022b).

Once the stem cell microenvironment/niche has been established, the maintenance and activation of the AM depend on a population of cells expressing the KNOX family genes viz., *SHOOTMERISTEMLESS* (*STM*) and *WUS* (Wang et al., 2014). For instance, it has been documented that the HD-ZIPIII family transcription factor REVOLUTA (REV) binds directly to the promoter of STM to upregulate its transcription (Otsuga et al., 2001). Concurrently, STM and WUS form a protein complex that acts as a core hub for maintaining the pluripotency of axillary meristematic cells and triggers AM formation (Su et al., 2020). Furthermore, the MYB transcription factor (RAX1) and the GRAS family member (LAS) are specifically expressed in the axillary microenvironment, and these genetic elements determine the ultimate fate of branches; for example, loss-of-function mutants of *RAX1* and LAS viz., *rax1* and *las*, respectively, show a strong reduction or complete absence of branches during the vegetative stage (Greb et al., 2003). The WRKY family transcription factor (EXB1) binds directly to the *RAX1* promoter to activate its expression, promoting a synergistic interaction between RAX1, LAS, and the bHLH transcription factor (ROX) to advance the AM formation (Guo et al., 2015). Furthermore, at the downstream hormonal feedback level, *LAS* exerts refined metabolic control i.e., it binds to and induces the expression of the gibberellin (GA) deactivation enzyme GA2ox4, thereby creating a local microenvironment with low GA concentration (Zhang et al., 2020). Reduced GA levels lead to the accumulation of DELLA proteins, which strongly inhibit the transcription factor SPL9; conversely, reduced SPL9 activity further promotes the transcriptional expression of *LAS*. This direct transcriptional activation mediated by *EXB1*, coupled with the downstream positive feedback metabolic loop involving *DELLA-SPL9-LAS-GA2ox4*, supports the spatiotemporal regulation of AM initiation (Zhang et al., 2020).

### 2.2 Regulation of axillary bud activation by hormonal crosstalk

Once the dormant axillary bud is formed, its ultimate fate i.e., whether it undergo rapid activation and elongation or it will remain dormant is determined by a complex endogenous hormonal network and the core branching repressor *BRANCHED1* (*BRC1*) (van Es et al., 2024). BRC1 encodes a TCP family transcription factor, which is specifically expressed in axillary buds and serves as the ultimate molecular hub for integrating and responding to diverse signals inhibiting branching (**Figure 2A**). Mechanistically, *BRC1* functions as a “growth brake” by directly binding to the promoters of its downstream targets *via* its highly conserved TCP domain, exerting dual transcriptional control. First, it directly inhibits genes associated with cell cycle progression and cell division, thereby blocking cell proliferation in the axillary meristem. Second, *BRC1* activates a series of genes involved in dormancy maintenance (such as the HD-ZIP transcription factors viz., HB21 and HB40), which efficiently induce the synthesis and accumulation of abscisic acid (ABA) in the local microenvironment, reinforcing axillary bud dormancy (González-Grandío et al., 2017).

**Figure 2:**
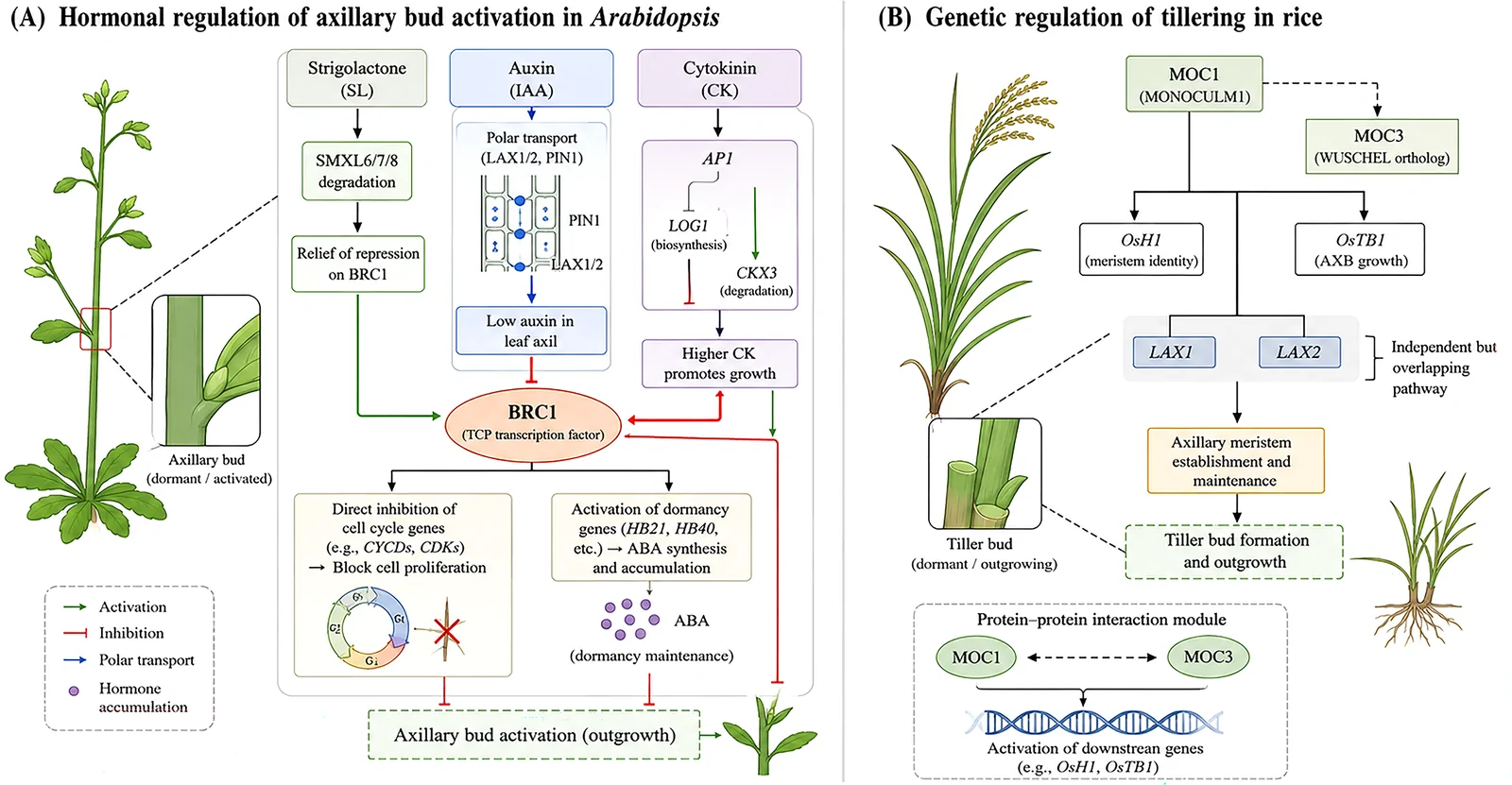
Regulatory networks controlling axillary bud activation in Arabidopsis and tillering in rice. **(A)** In Arabidopsis, strigolactone (SL) promotes the degradation of SMXL6/7/8, relieving their repression on BRC1 and leading to its accumulation. BRC1 directly inhibits cell cycle–related genes to block cell proliferation and activates dormancy genes (*HB21, HB40*) to induce local ABA accumulation, thereby maintaining bud dormancy. Polar auxin transport mediated by LAX1/2 and PIN1 maintains low auxin levels in the leaf axil, which relieves BRC1-mediated repression. Cytokinin (CK) acts antagonistically to auxin; AP1 suppresses CK biosynthesis gene *LOG1* and activates CK degradation gene *CKX3*, shaping local CK homeostasis to promote bud outgrowth. **(B)** In rice, MOC1 is the central regulator that activates *OsH1* and *OsTB1* and interacts with MOC3 (a WUSCHEL ortholog). Together with *LAX1* and *LAX2*, MOC1 establishes and maintains axillary meristems, ultimately leading to tiller bud formation and outgrowth.

Through these synergistic effects of *BRC1* viz., “physical blockade of the cell cycle” and “chemical induction of dormancy hormones”; this gene translates systemic inhibitory signals into the developmental decision to arrest bud growth. In the hormonal network of regulating axillary bud activation and lateral growth, the auxin (IAA) is produced mainly in the shoot apex and transported basipetal. This establishes apical dominance, in which the main shoot suppresses the growth of axillary buds. For instance, studies have documented that mutation or downregulation of key genes involved in the local IAA biosynthetic pathway within axillary buds (e.g., *YUC* and *TAA1*) reduces IAA synthesis thereby breaking apical dominance leading to a flush of lateral branching (Mashiguchi et al., 2011; Liu et al., 2024). The maintenance of low IAA levels in the leaf axils depends on precise polar transport mediated by the influx carrier *LAX1/2* and the efflux carrier *PIN1*. In contrast, cytokinin (CK) acts antagonistically to auxin (IAA) in the regulation of shoot branching by promoting axillary bud activation and counteracting auxin-mediated apical dominance (Müller and Leyser, 2011; Liu et al., 2024). Through stimulation of axillary meristem activity and cell proliferation, CK facilitates subsequent lateral shoot development and branch elongation (Müller and Leyser, 2011; Liu et al., 2024). In Arabidopsis, defects in AM initiation in *rax* mutants can be partially rescued by exogenous CK or by ectopic expression of the CK biosynthesis gene *IPT8*, supporting a role for CK in promoting AM initiation (Wang et al., 2014). In a distinct developmental context, Han et al. (2014) demonstrated that the floral meristem identity factor *APETALA1* (*AP1*) directly represses the CK biosynthesis gene *LOG1* and activates the CK degradation gene *CKX3*, thereby reducing CK levels in developing floral tissues and restricting secondary floral meristem formation in sepal axils. Thus, the AP1–LOG1–CKX3 module provides evidence for transcriptional regulation of CK homeostasis during floral-meristem determinacy, but should not be interpreted as direct evidence that this module controls vegetative axillary-bud activation or shoot-branch outgrowth. The strigolactone (SL) acts as a negative regulator for axillary bud elongation, and this hormone does so through a highly conserved ubiquitin-mediated degradation mechanism (Dun et al., 2023). The SLs are perceived by the D14 receptor, which upon ligand activation forms a complex with the SCF-MAX2 E3 ubiquitin ligase complex, leading to ubiquitination and proteasomal degradation of SMXL/D53 repressors and activation of downstream SL signaling (Jiang et al., 2013; Liu et al., 2024). In Arabidopsis, SMXL6, SMXL7, and SMXL8 function as major SL signaling repressors and are direct targets of D14-SCF-MAX2-mediated degradation (Soundappan et al., 2015). Hence, upon SL perception, D14–SCF-MAX2-mediated degradation of SMXL6/7/8 activates SL signaling, that in turns promotes *BRC1* expression and maintaining axillary buds in an inactive state by suppressing their outgrowth (Soundappan et al., 2015; Liu et al., 2024). Furthermore, it has been revealed that SL can induce reticulon-mediated endocytosis of *PIN1*, which depletes membrane-localized *PIN1* and thus blocks polar auxin transport in the main stem, establishing a molecular crosstalk between the SL and IAA pathways (Shinohara et al., 2013; Liu et al., 2024). Overall, the combination of auxin-mediated inhibition, cytokinin-promoted bud outgrowth, and strigolactone-mediated repression constitutes a precise spatiotemporal molecular network governing the axillary bud outgrowth in model plant of Arabidopsis.

Furthermore, the plant density-dependent regulation of genes involved in axillary bud development, hormonal signaling and excessive vegetative growth has been documented in Arabidopsis (Holalu et al., 2020). For instance, increased planting density alters the canopy light environment, particularly by decreasing the red-to-far-red (R) ratio. This signal is perceived primarily by phytochrome B (phyB), and reduced phyB activity promotes the activity of PHYTOCHROME-INTERACTING FACTORS (PIFs), particularly PIF4 and PIF5. These transcription factors promote the expression of *BRC1* in axillary buds, thereby suppressing bud outgrowth and reducing shoot branching (Holalu et al., 2020).

## 3 Genetic basis and regulatory pathways of rice tillering

Rice (*Oryza sativa*) is the model for monocotyledonous species, that possesses a branching system known as “tillering.” The number of tillers directly determines number of effective tillers bearing panicles, thereby governing the final grain yield. Although the mechanisms of rice tillering share logical similarities with those in Arabidopsis, rice has evolved distinct genetic modules controlling key regulatory nodes (**Figure 2**).

### 3.1 Key genetic factors and interactions regulating tiller bud formation

In rice, the initiation and early development of AM are governed by the core GRAS family transcription factor viz., MONOCULM1 (MOC1). Studies have documented that the loss-of-function mutant *moc1* possessed a single culm with a complete absence of tillers, whereas overexpression of *MOC1* leads to a significant increase in the number of axillary buds as well as tillers (Li et al., 2003). Moreover, it has been well demonstrated that *MOC1* regulates the expression of *OsH1* (a key factor involved in maintaining meristem identity) and *OsTB1* (a key regulator involved in axillary bud growth as well as the ortholog of Arabidopsis *BRC1*), thereby revealing the molecular mechanism by which MOC1 regulates AM initiation (Li et al., 2003; Takeda et al., 2003).

Following the initial formation of AM, its further development and maintenance of AM are governed by LAX1 (encodes a basic helix-loop-helix/bHLH transcription factor) and LAX2 (which encodes a plant-specific nuclear protein that acts as a novel co-factor). The *LAX2* interacts physically with the *LAX1* to control AM development (Tabuchi et al., 2011). These authors showed that the *lax1lax2* double mutant synergistically enhance the reduced-branching phenotype. Tabuchi et al. (2011) demonstrated that *LAX1* and *LAX2* function through a pathway partially independent of, yet functionally overlapping with *MOC1* to drive the subsequent development and long-term maintenance of the AM. *MOC1* is known to physically bind with *MOC3*, which is an ortholog of the Arabidopsis meristem regulatory hub gene *WUSCHEL*. *MOC3* possesses strong transcriptional activation activity and binds to the promoter regions of critical downstream genes. Its expression is induced by cytokinin, placing it at a vital intersection where phytohormone signaling drives plant architecture (Shao et al., 2019). Within this complex, *MOC1* acts as a core co-activator for *MOC3*, and their synergistic action significantly enhances the transcription of downstream targets such as the *FLORAL ORGAN NUMBER 1* (*FON1*), which specifically regulates tiller bud formation and outgrowth (Shao et al., 2019).

Furthermore, to prevent the excessive depletion of nutrients resulting from unrestricted meristematic activity, rice has evolved a sophisticated negative feedback module involving ubiquitin-mediated degradation. The co-activator of the multi-subunit E3 ubiquitin ligase (APC/C) viz., TAD1, specifically recognizes and interacts with MOC1 and OsAPC10 to form a complex that ubiquitinates MOC1 and targets it for proteasomal degradation in a cell-cycle-dependent manner (Xu et al., 2012). This *MOC1*-centered regulatory framework, comprising transcriptional cascades, complex activation, and TAD1-mediated precise protein degradation, spatially and temporally restricts excessive tiller bud proliferation, ensuring balanced plant architecture development (Xu et al., 2012).

### 3.2 Molecular hubs and networks governing tiller bud elongation

Once tiller buds are formed, their developmental fate viz., outgrowth versus continued dormancy is determined by highly specialized transcriptional regulatory hubs and hormonal networks. At this stage, rice relies heavily on the core regulator *OsTB1/FC1* (an ortholog of Arabidopsis *BRC1*) (**Figure 2B**) (Takeda et al., 2003). *OsTB1* encodes a TCP family transcription factor, and this gene is specifically expressed in axillary buds and acts as a “brake” on lateral bud development. Genetic studies have revealed its precise role in shaping plant architecture. Frameshift mutations in *OsTB1* significantly increase tiller number, resulting in a 3.5-fold increase in panicle numbers compared to the wild-type plants at maturity (Ishizaki et al., 2023). This study also reported that higher tillering produced more spikelets; however, there was no overall enhancement in grain yield, especially due to decreased grain filling rate and grain weight (Ishizaki et al., 2023). Conversely, non-frameshift mutations can effectively increase panicle number and yield under phosphorus-deficient conditions, and the natural allele *MP3* has been shown to significantly enhance rice productivity under elevated atmospheric CO_2_ concentrations (Takai et al., 2023; Yang et al., 2024).

The SQUAMOSA promoter-binding protein viz., *Ideal Plant Architecture1* (IPA1) or OsSPL14 binds directly to the *OsTB1* promoter, thereby regulating its expression at the transcriptional level. This pathway is of paramount importance in the breeding of “ideal plant architecture” in rice. For instance, the *IPA1* directly upregulates expression of *OsTB1* to suppress axillary bud outgrowth, effectively reducing tiller number (Wang et al., 2020). The *qWS8/ipa1-2D* locus is a natural variation in rice where three tandem repeats occur in the promoter region upstream of the *IPA1* gene (Zhang et al., 2017). This specific structural variation alters the epigenetic state of the plant (Zhang et al., 2017). Specifically, it significantly reduces DNA methylation levels in the *IPA1* promoter region, leading to an open chromatin structure and effectively releasing the epigenetic repression imposed by heterochromatin. Such epigenetic remodeling dramatically unleashes the expression potential of *IPA1*, which in turn optimizes rice architecture and grain yield by precisely controlling downstream targets like *OsTB1* (Jiao et al., 2010; Zhang et al., 2017). For example, Zhang et al. (2017) revealed that *IPA* traits can be fine-tuned by manipulating *IPA1* expression and that an optimal *IPA1* expression level may lead to an ideal yield, demonstrating a practical approach for designing elite super rice varieties.

Similar to Arabidopsis, rice tiller elongation is strictly governed by the systemic SL pathway. In the rice SL signaling chain, the receptor D14, the F-box protein D3, and the signaling repressor D53 form the core transduction complex. Upon perception of SL, the D14-D3 complex targets D53 for ubiquitin-mediated degradation, directly abolishing D53’s activity in promoting axillary bud growth, which results in a sharp reduction in tiller number (Jiang et al., 2013; Zhou et al., 2013; Liu et al., 2024). This pathway also exhibits deep crosstalk with the brassinosteroid (BR) signaling pathway; both SL and BR act antagonistically on the D53-OsBZR1 protein complex to precisely determine the final abundance of *OsTB1/FC1* (Fang et al., 2020). Research has further shown that the SL pathway is directly coupled with environmental nutrient signals. Under low-phosphorus stress, the core rice phosphorus-signaling regulator *OsPHR2* is rapidly activated, directly inducing the transcription of nodulation signaling pathway genes *NSP1* and *NSP2* (Yuan et al., 2023). Subsequently, *NSP1* and *NSP2* form a heterodimer that strongly upregulates the expression of downstream SL biosynthetic genes (*D10*, *D17*, and *D27*). This cascade leads to a dramatic increase in SL levels in the roots, which in turn significantly promotes the expression of the negative regulator *OsTB1*, thereby inhibiting tiller bud elongation and allowing the plant to reallocate resources to adapt to nutrient-poor environments (Liu et al., 2011; Yuan et al., 2023). In addition, the protein kinase gene *FON1*, which functions independently of the AM initiation process, acts as a specific positive regulator of tiller bud elongation. Mutation of *FON1* results in a significant reduction in tiller elongation, whereas the upstream MOC1-MOC3 complex drives tiller bud development by enhancing *FON1* expression (Moon et al., 2006; Shao et al., 2019).

## 4 Genetic regulatory networks in soybean branching number

The architecture of soybean, particularly the number of branches directly determines canopy closure, light interception, and ultimately, effective pod number and grain yield. However, due to the complexity of the soybean genome, the molecular genetic characterization of branching traits has lagged behind that of Arabidopsis and rice. Recently, with the rapid advancement of functional genomics, the mysteries surrounding the regulatory mechanisms of soybean branching have gradually become clearer, providing new opportunities for molecular breeding and plant architecture improvement (**Table 1**).

**Table 1:** Summary of some identified genes related to shoot branching in plants.

Species	Regulatory module	Gene/QTL name	Core function described in this review	References
*A. thaliana*	AM initiation & maintenance	*CUC2/CUC3*	Prerequisites for boundary specification and AM induction must be repressed as development progresses.	Li et al. (2020)
*A. thaliana*	AM initiation & maintenance	*LAS*	Induces *GA2ox4* to create a low-GA microenvironment; mediates the *DELLA-SPL9* positive feedback loop.	Zhang et al. (2020)
*A. thaliana*	AM initiation & maintenance	*STM/WUS*	Continuously expressed to maintain meristematic cell activity; transcription is directly upregulated by *REV*.	Wang et al. (2014)
*A. thaliana*	Hormone response & elongation	*BRC1*	A TCP transcription factor that serves as the final integration hub for diverse branching-inhibitory signals (e.g., SL).	van Es et al. (2024)
*A. thaliana*	Hormone response & elongation	*IPT8/AP1*	*IPT8* promotes cytokinin (CK) biosynthesis; *AP1* maintains CK homeostasis via *LOG1* and *CKX3* to promote branching.	Han et al. (2014)
*O. sativa*	Tiller bud formation	*MOC1/MOC3*	*MOC1* is the core factor; forms a complex with *MOC3* to synergistically enhance downstream target gene expression.	Li et al. (2003)
*O. sativa*	Tiller bud formation	*TAD1*	Identifies and interacts with *MOC1* to guide its degradation, spatially and temporally restricting tiller proliferation.	Xu et al. (2012)
*O. sativa*	Elongation & architecture	*OsTB1*	Ortholog of *BRC1*; specifically expressed in axillary buds to act as a “brake” on lateral bud development.	Takeda et al. (2003)
*O. sativa*	Elongation & architecture	*IPA1 (OsSPL14)*	Directly binds to the *OsTB1* promoter; critical for optimizing panicle number and “ideal plant architecture.”	Zhang et al. (2017)
*O. sativa*	Elongation & architecture	*D14-D3-D53*	SL-sensing complex; targets the signaling repressor *D53* for ubiquitin-mediated degradation to inhibit tillering.	Jiang et al. (2013)
*G. max*	Architecture & breeding	*Dt2*	Dominant transcription factor coupled with the transition to reproductive growth; suppresses branching via *GmApla* and *GmApld*.	Liang et al. (2022)
*G. max*	Architecture & signaling	*GmSPL8/9*	miR156-targeted SBP-box TFs; integrate age and photoperiod signals to regulate the vegetative-to-reproductive transition.	Sun et al. (2019)
*G. max*	Elongation & architecture	*GmBRC1*	CYC/TB1-type TCP transcription factor proposed as a negative regulator of soybean branching; its expression is associated with differences in branch number, particularly in the shoot apex.	Shim et al. (2019)
*G. max*	Elongation & architecture	*GmTB1*	TB1/CYC-type TCP transcription factors homologous to maize TB1; associated with suppression of axillary bud outgrowth and branch development.	Tang et al. (2026)
*G. max*	AM/tiller bud initiation	*GmMOC1-*like genes	Putative soybean homologs of rice MOC1 identified through genome-wide homology; potentially associated with axillary meristem/tiller-bud initiation, but direct functional validation in soybean remains limited.	Schmutz et al. (2010)
*G. max*	Hormone response & elongation	*GmMAX/D14/D3*	Conserved strigolactone biosynthesis and signaling components; GmMAX genes participate in SL biosynthesis, while GmD14/GmMAX2 represent components of SL perception/signaling that regulate shoot branching.	Haq et al. (2017); Qiao et al. (2020)
*G. max*	Cytokinin-mediated branching	*GmTMS6–GmCKX*	GmTMS6 represses branching by activating CKX genes in axillary buds, promoting cytokinin degradation and thereby restricting axillary bud outgrowth.	Gu et al. (2026)

### 4.1 Genetic and molecular networks regulating branching number in soybean

In legumes, relatively limited efforts have been devoted to elucidate the functional genes and molecular networks regulating the branch number. However, significant progress has recently been achieved in soybean to decipher the genetic and molecular networks underlying soybean plant architecture. Soybean homologs of major branching genes viz., *GmBRC1* (Shim et al., 2019), *GmTB1* (Tang et al., 2026), *GmMOC1*-like genes (Yan et al., 2020), *GmMAX/D14/D3* strigolactone pathway components (Haq et al., 2017; Qiao et al., 2020), and cytokinin-related regulators such as *GmTMS6–GmCKX* module (Gu et al., 2026) have been identified; however, compared to rice and Arabidopsis, their regulatory networks and functional interactions in soybean are not comprehensively characterized. Moreover, the important question has been unresolved that is whether soybean utilizes conserved regulatory modules similar to rice tillering or Arabidopsis axillary branching, or whether legume-specific mechanisms have evolved to control branching architecture. In order to translate knowledge from model species into soybean ideotype design and breeding for optimal plant architecture, it will be crucial to integrate comparative genomics, transcriptomics, genome editing, and hormone-responsive phenotyping.

The dominant gene Dt2 has been identified as a core factor negatively regulating branch number in soybean (Liang et al., 2022). Dt2 is primarily known for regulating the developmental transition from vegetative to reproductive growth in soybean. At the molecular level, the transcription factor *Dt2* interacts with *GmAgl22* and *GmSoc1a* to physically bind to the promoters of *GmAp1a* and *GmAp1d*, thereby activating their transcription (Liang et al., 2022; Li W. et al., 2024). *GmAp1* functions as a positive regulator in the transition from vegetative growth into reproductive growth by promoting the transition of AM into the inflorescence meristem (IM). *Dt2* interacts with *GmAgl22* and *GmSoc1* to activate the transcription of *GmAp1*. Furthermore, the *Dt2*^HapII^ promoter has higher transcriptional activity than the *Dt2*^HapI^ promoter, which in turn enhances the expression of *GmAp1* and promotes earlier transition from vegetative growth to reproductive growth, resulting in earlier flowering and reduced branching (Liang et al., 2022). Consistent with these findings, the Arabidopsis homologs of *Dt2*-*GmSoc1a*, *FUL*-*SOC1*, were also found to regulate branch number (Melzer et al., 2008; Bemer et al., 2017; Karami et al., 2020). Therefore, by fine-tuning the transcriptional expression of these targets, *Dt2* regulates apical dominance and effectively suppresses lateral axillary bud outgrowth (Liang et al., 2022; Zhang et al., 2019).

In addition to the *Dt2*-mediated direct repression pathway, the SBP-box family transcription factors SPL, which are targeted by microRNA (*miR156*), constitute another highly specific genetic mechanism in the soybean for the regulation of shoot branching (Bao et al., 2019). It has been reported that transgenic soybean plants overexpressing *GmmiR156b* exhibit significantly altered plant architecture, leading to a substantial increase in grain yield per plant (Sun et al., 2019). Furthermore, previous studies have reported that the *GmSPL9d* gene is expressed in both the stem apical meristem (SAM) and the axillary meristem (AM) of soybeans; moreover, *GmSPL9d* may regulate axillary bud formation and stem meristem development by engaging in a physical interaction with WUS (Sun et al., 2019). Based on these findings, Bao et al. (2019) utilized CRISPR/Cas9 technology to validate the SPL9 gene family in soybeans and discovered that introducing mutations into four *SPL9* genes resulted in altered plant morphology in soybean plants. Shim et al. (2019) used the integrated approach of GWAS and linkage mapping, and identified the *GmBRC1* as a candidate gene for regulating branching in soybean. Ji et al. (2025) demonstrated that erect growth habit in cultivated soybean reduces cytokinin content and restricts basal branching; exogenous cytokinin promotes this branching. *GmBRC1*, expressed specifically in cotyledonary and node 1 buds, is repressed by cytokinin. Collectively, the *Dt2*-dominated transcriptional regulatory pathway, the *miR156-GmSPL9* module-mediated pathway and cytokinin-GmBRC1 module-mediated operate in a synergistic and parallel manner, together constructing the highly specific logic of soybean branch development. The elucidation of these core mechanisms provides precise genetic targets for the directed modification of soybean architecture and facilitates the implementation of molecular design breeding.

### 4.2 Genetic factors identified for soybean branching number

Branch number is a complex quantitative trait in soybean, regulated by multiple genes/QTLs/haplotypes as well as is also strongly influenced by the environment (Yu et al., 2024; Li W. et al., 2024). Traditional linkage mapping has identified 21 branching-related QTLs recorded in SoyBase (https://www.soybase.org/).; however, these loci suffer from low resolution, large confidence intervals and insufficient cross-genetic-background validation, severely restricting their breeding application. As an advanced alternative, GWAS combined with haplotype analysis overcomes these limitations (**Table 2**). For instance, Yu et al. (2024) identified seven high-resolution QTLs (*qBN7*, *qBN12*, *qBN13*, *qBN16*, *qBN18*, *qBN19*, *qBN20*) with refined intervals; *qBN12* further narrowed the physical region of the previously reported *Branching* 5–3 locus (Shim et al., 2017). They also detected eleven haplotype blocks containing 2–5 alleles with divergent effects on branching traits, offering rich genetic resources for plant architecture improvement, albeit requiring phenotypic validation across genetic backgrounds.

**Table 2:** List of markers associated with the shoot branch number in soybean.

Marker/QTL	Marker type	Current evidence	Classification for breeding	References
*GmBRC1*	Gene-specific SNP	Association + expression + NIL valuation	Candidate gene-based markers	Shim et al. (2019)
*qBN-1*	BARCSOYSSR_06_1048/ BARCSOYSSR_06_1053	Fine-mapped to 115.67 kb	Tightly linked markers	Lamlom et al. (2020)
*qBN-18*	BR69, BR77 (InDel)	Fine-mapped to 63.7 kb	Tightly linked markers	Wu et al. (2020)
*qBN7*	SNP around Chr07_29399935	Stable across environments/models	Candidate association marker	Yu et al. (2024)
*qBN12*	SNP around Chr12_5601871	Stable QTL across environments	Candidate/tightly associated marker	Yu et al. (2024)
*qBN13*	SNP around Chr13_17432336	Multi-environment GWAS	Candidate association marker	Yu et al. (2024)
*qBN16*	SNP around Chr16_6096184	Multi-environment GWAS	Candidate association marker	Yu et al. (2024)
*qBN18*	SNP around Chr18_32068331	Multi-environment GWAS	Candidate association marker	Yu et al. (2024)
*qBN19*	SNP around Chr19_11321785	Multi-environment GWAS	Candidate association marker	Yu et al. (2024)
*qBN20*	SNP around Chr20_18794801	Multi-environment GWAS	Candidate association marker	Yu et al. (2024)
*qGmBa1*	QTL-region markers	Detected in two populations including SoyNAM	Tightly linked/MAS candidate	Clark et al. (2022)
Multiple GWAS SNPs	SNP	Association studies	Candidate markers	Virdi et al. (2023)
Multiple GWAS SNPs	SNP	Multi-environment association	Candidate markers	Virdi et al. (2023)
*qBN12*	SNP	Multi-environment GWAS	Candidate association marker	Shim et al. (2017)
*qNVB-1-1*	SSR	Linkage-based QTL	QTL-linked marker	Yao et al. (2015)
*qBR6-1*	SNP/QTL	Major QTL for branch number	Stable QTL marker	Shim et al. (2017)
*qBR11-1*	SNP/QTL	QTL associated with branch number	QTL-linked marker	Shim et al. (2017)
*qBR19-1*	SNP/QTL	QTL associated with branch number	QTL-linked marker	Shim et al. (2017)
*GmBRC1/Glyma.06G210600*	Functional gene/SNP	GWAS, expression, and natural-variation evidence	Functional gene marker	Shim et al. (2019)
*qGmBN18-1*	GWAS-SNP	SoySNP50K association	Candidate association marker	Virdi et al. (2023)
*qGmBA19-1*	GWAS-SNP	SoySNP50K association; overlaps with a canopy-coverage QTL	Candidate association marker	Virdi et al. (2023)
*qGmBa1*	SNP/QTL	Detected in both biparental and SoyNAM populations	Stable QTL candidate marker	Clark et al. (2022)
*Dt2*	Functional gene/natural variation	Natural variation associated with branching phenotype	Functional gene marker	Liang et al. (2022)

Soybean shoot branching is a complex quantitative trait controlled by multiple QTLs with pleiotropy, tight linkage and epistatic interactions (Guang et al., 2017; Sayama et al., 2010). Branching-related QTLs co-localize with flowering-time loci, and the phenotypic effect of branching loci depends on genetic background, indicating branching is regulated by both additive QTL effects and inter-locus epistasis (Sayama et al., 2010; Guang et al., 2017). Multiple QTL hotspots for branching and plant architecture traits have been identified on several soybean chromosomes (Yang et al., 2025; Wang et al., 2026), which are valuable breeding targets, while allelic effects and QTL-by-environment interactions require further verification. Yang et al. (2025) performed the high-density QTL analysis for four branching-related traits viz., branch number, branch internode number, branch length and branch internode length, in soybean; and have detected a total of 251 QTLs associated with these four branching-related traits, and these QTLs are harbored within 143 genomic regions. Moreover, Wang et al. (2026) performed QTL mapping analysis in multiple environments, and detected co-localization of QTLs associated to various traits viz., branch number, bottom-pod height, plant height and main-stem node number, including stable genomic regions on Chr.06, Chr.10 and Chr.19; and some loci showed QTL × environment interactions. Hence, these pleiotropy loci and QTL hotspots associated with multiple architectural traits can serve as the important targets in the marker-assisted breeding for the simultaneous improvement of plant architecture in soybean through the selection of favorable alleles influencing multiple correlated traits.

Recently, pangenome studies reveal that structural variations (SVs) and presence/absence variations (PAVs) substantially influence agronomic traits and complement SNP-based genetic analysis (Liu et al., 2020; Li et al., 2026; Yano et al., 2025). The key branching gene *GmBRC1* is a critical target for SV analysis (Shim et al., 2019).

### 4.3 Translational research for breeding branching in soybean

As discussed above, in the model plant species such as Arabidopsis and rice the genetic and molecular mechanism has been deeply elucidated. The maintenance of meristematic competence and subsequent axillary meristem initiation is regulated by a set of transcription factors (TFs), which are conserved between species. Therefore, it is feasible to systematically present the cross-species genetic principles of lateral branching and the specific targets for soybean breeding. Integrating information from Arabidopsis, rice, and soybean enables the identification of conserved regulatory modules as well as soybean-specific targets for molecular breeding (**Tables 1**, **2**). This multi-dimensional genetic roadmap provides a theoretical framework for subsequent gene pyramiding and cross-scale molecular design breeding.

The molecular markers, genes and QTLs found associated with branching-related traits represent the valuable genomic resource that can be used for the development of high-yielding soybean cultivars with optimized plant architecture. The functional genes and major-effect QTLs validated in multiple genetic backgrounds can be used by the breeder in the MAS to introgress favorable/desirable/superior alleles into elite soybean cultivars. However, the soybean branching-related traits are complex traits governed by polygenes, thus genomic selection (GS) is the best strategy that can use the genome-wide marker profile to predict the breeding values of soybean accessions/cultivars, thereby speed-up the accumulation of favorable allelic combinations in the germplasm or cultivars (Bhat et al., 2021). Besides, using haplotype-based selection across important genomic regions will facilitate the detection of superior allele combinations linked with optimized branching architecture such as suitable branch number, branch angle and branch nodes allocation (Bhat et al., 2021; Yu et al., 2024). The GWAS-based identification of candidate genes with their appropriate functional validation can be suitable target for further genome editing to adjust the gene expression or allelic dosage (Yu et al., 2024). However, branching-related genes require the validation across diverse genetic backgrounds and environments before they can be deployed in the molecular breeding for the development of cultivars with optimized branching architecture; it is because the branching-related genes often show pleiotropic effect on other agronomic traits (Li J. et al., 2024). However, it is suggested that combined use of the marker-assisted breeding approaches (such as MAS and GS) with the GWAS-derived markers, haplotype information, and high-throughput phenotyping in multiple environments provides a holistic framework for gaining the pace in the development of high-yielding climate-resilient soybean cultivars with desirable branching architecture.

It is evident that optimized branching architecture is the core objective of the soybean breeders for maximizing the yield potential; however, the individual branching traits/architectural traits are genetically correlated with other agronomic traits in soybean, therefore selection based on single branching trait do not always give superior cultivars (Li W. et al., 2024; Hou et al., 2026). For instance, cultivars possessing the phenotype of excessive branching will have increased vegetative biomass but have negative effect on the other agronomic traits viz., lodging resistance, maturity, canopy architecture or reproductive nodes distribution. Consequently, a balanced multi-trait selection approach that concurrently takes into account branching traits, yield components, plant height, maturity, and stress adaptation should be used in breeding programs (Li W. et al., 2024; Fang et al., 2024). Besides, genotype × environment (G × E) interactions may lead to variations in the expression and contribution of branching-related genes and QTLs across environments. Hence, it is prerequisite to evaluate the candidate alleles and superior haplotypes in multiple years and environments to verify their stability in the phenotype determination (Yu et al., 2024). Furthermore, since plant population density has a significant impact on light competition, resource availability, and compensatory branching responses, thus genotype × planting-density interactions is important to be taken into consideration (Agudamu et al., 2016). For instance, a soybean cultivar possessing desirable branching architecture is performing better in the low-density conditions, but it is not necessary that this cultivar shows the same performance under the high-density conditions. Therefore, it is essential to integrate multi-environment trials, density-specific phenotyping, and genomic data for developing soybean cultivars with stable and optimal branching architecture under diverse production systems.

## 5 Comparison of agronomic performance of varied branching number in soybean

### 5.1 Shoot branching number associated agronomic traits

Soybean canopy structure, pod distribution, and suitability for mechanical harvesting are all strongly impacted by shoot branching features, such as branch number, branch angle, and branch position (Sreekanta et al., 2024). The cultivars with higher-branching phenotyping frequently produced more reproductive nodes and enhanced pod number; however, the development of branches at the lower positions of the main stem decreases the first-pod height, thereby the number of pods close to the soil surface has considerable increases, which in turn leads to the greater losses of the overall yield due to the incomplete cutting by combine harvesters (Ferreira et al., 2018; Rębilas et al., 2020). On the other hand, cultivars possessing more/optimized first-pod height and well-positioned branches will prevent the losses occurring through mechanical harvesting systems (Ramteke et al., 2012; Virdi et al., 2023). There are also the interactions of the branch architecture with the stem growth and plant height; for instance, increased growth of main-stem will increase the first-pod height thereby enhances the risk of lodging; on the other hand, the higher compact architecture of the plants might lead to decreased canopy development as well as reduced yield potential (Hou et al., 2026). Therefore, breeding for mechanized soybean production requires an optimal plant architecture that combines suitable first-pod insertion height, appropriate branch number and angle, moderate plant height, high lodging resistance, and effective distribution of reproductive nodes. Integration of branching-related genes/QTLs with phenotypic selection for harvestability features may allow the production of soybean cultivars that maintain high yield while minimizing mechanical harvest losses.

### 5.2 Agronomic performance of varied branching number

Soybean cultivars with contrasting branching patterns possesses different merits in varied environments and management systems. For example, soybean cultivars possessing higher branching pattern produces more nodes, pods and seed/plant, hence under low-density planting it contributes to yield compensation (Agudamu et al., 2016). But the higher number of excessive branches led to increased vegetative growth, higher intensity of intra-plant competition, and the reduced efficiency of light distribution across the dense canopies (Li N. et al., 2025). In contrast, the soybean cultivars with compact low-branching pattern revealed enhanced adaptation to high-density planting systems especially through reduced inter-plant competition, rapid closure of canopy, and maintaining higher yield stability via producing more pods per unit area as opposed to per plant (Fang et al., 2026). It has been demonstrated that soybean cultivars with higher ability of branching can recompense decreased plant population under sparse planting, while as low-branching cultivars under high-density planting often perform better because of enhanced productivity of population (Xu et al., 2021). Hence, it is evident that higher or lower branching cannot define the ideal architecture of soybean, but an optimized architecture is required that makes balance between branch number, productivity of main stem, resistance to lodging, structure of canopy, and distribution of reproductive structures. Many factors affect the yield contribution made by the branches, and these factors include type of genotype, density of planting and environmental conditions; thus, emphasizing the significance of choosing branching ideotypes that complement particular target environment and cultivation strategies.

### 5.3 Comparative perspective for major soybean-producing regions

Regional production strategies, including planting density, row spacing, maturity group, and environmental factors, have a significant impact on the optimal branching architecture of soybeans. Therefore, it is prerequisite to optimize branch number and canopy architecture in accordance to specific agroecological environments for balancing yield per plant and overall yield (Li W. et al., 2024; Li R. et al., 2025). For instance, the majority of Brazil’s soybean production is based on indeterminate and semi-determinate cultivars that are adapted to tropical and subtropical climates, where moderate plant populations and relatively wide row spacing (about 40–50 cm in many systems) allow for greater expression of branch development and contribute to enhanced yield through increased pod production on branches (Ferreira et al., 2018; 2019). Moreover, research conducted in Brazilian production systems has shown that plant density and arrangement affect how branches contribute to grain yield, with lower densities typically encouraging more branching and compensating yield formation per plant (Ferreira et al., 2018; Balbinot Junior et al., 2018). On the other hand, the soybean production in the United States of America (USA) especially in the Midwest, often utilizes narrow-row system (≤38 cm) and diverse maturity groups suited to various latitudes; and the cultivars used for planting possess reduced branching, compact architecture and enhanced main-stem yield that have merits of efficient closure of canopy and high-density production (De Bruin and Pedersen, 2008; Thompson et al., 2015). The soybean production in China is carried out across diverse agroecological zones ranging from the Northeast China with high latitude to the southern parts which usually involves subtropical and tropical regions; this wide variation in the environments have led to the development and allocation of soybean cultivars with different photoperiod adaptations, maturity groups and growth habits suited to particular agroecological zones (Wu et al., 2023). However, recent efforts made in the China have largely focused in the development of cultivars with optimized plant architecture, that are emphasizing on compact canopy, well-distribution of light and optimized branching pattern to increase the yield under intensive management systems (Li W. et al., 2024; Li R. et al., 2025). These regional variations suggest that a single branching ideotype might not be the best option for all situations; instead, breeding goals should take local production environments into account. Soybean cultivars that have low-branching and compact phenotype are suitable for the high-density planting, whereas cultivars with highly branched phenotype are suitable for low density planting. Therefore, developing soybean cultivars with optimal branching architecture and stable yield performance will need integration of regional agronomic practices with genomic selection and multi-environment testing.

### 5.4 Challenges and strategies for breeding soybean branching number

Despite preliminary successes in the identification of some genes for soybean branching, achieving the comprehensive molecular design of an “ideal” soybean architecture remains a major scientific challenge. Some of the challenges soybean branching is facing are as follow; (1) genomic complexity and high redundancy: Soybean possesses a massive and highly complex genome, which has undergone multiple whole-genome duplication (WGD) events throughout the soybean evolution. This has resulted in extensive functional redundancy among homologous copies of branching-related genes. Hence, single-gene knockouts often fail to produce distinct phenotypic changes, vastly complicating the validation of gene function (Schmutz et al., 2010; Fang et al., 2023; Tian et al., 2025). (2) bottlenecks in genetic transformation and tissue culture: Soybean is widely regarded as a “recalcitrant” crop for transformation. The extreme difficulty in legume tissue culture and low regeneration efficiency severely limits the pace of functional genomics research. Even when potential branching-regulatory genes are cloned, the low efficiency of genetic transformation hinders the rapid generation of corresponding mutant materials for phenotypic analysis (Xu et al., 2022; Tian et al., 2025). (3) Phenotypic instability due to environmental sensitivity: Soybean is a classic short-day plant, and branching initiation is tightly linked to the photoperiod pathway and flowering time (Liang et al., 2025). The effect of a specific branching gene may fluctuate significantly across different latitudes or planting dates. Decoupling the genetic linkage between flowering time and branch number, while maintaining regional adaptability, remains an urgent challenge in soybean breeding (Li W. et al., 2024; Liang et al., 2025).

To address these challenges, the future of soybean branching trait improvement must rely on the deep integration of multidisciplinary approaches and disruptive frontier breeding technologies (**Figure 3**). (1) Precision intervention via CRISPR/Cas genome editing: With the development of advanced genome-editing technologies, including CRISPR/Cas9 and CRISPR/Cas12, researchers can complement conventional breeding by introducing targeted genetic modifications to genes or regulatory regions underlying important agronomic traits (Bai et al., 2024; Li R. et al., 2025). These tools allow for direct, site-specific knockout or promoter modification of the *Dt2* gene cluster or newly discovered branching repressors, enabling the “customization” of branching numbers in soybean (**Figure 3A**) (Bao et al., 2019; Zhang et al., 2019; Li N. et al., 2025). (2) Revolutionary integration of the haploid induction system (HIS). To overcome the long homozygous cycle of traditional soybean breeding (typically requiring 6–8 generations), emerging gene-editing-mediated HIS exhibit enormous potential. By targeting and editing key conserved genes involved in haploid induction (e.g., *GmDMP*), researchers can develop soybean-specific maternal haploid inducer lines. Integrating CRISPR technology with HIS can allows the rapid acquisition of double-haploid (DH) edited soybean lines/germplasm that are characterized by precise gene edits and complete homozygosity within few generations, thus representing a future promising strategy for accelerating soybean architecture improvement (**Figure 3B**) (Wang et al., 2019; Liu et al., 2025; Musazade et al., 2025). (3) Multi-omics integration for regulatory network deciphering: Transcriptomics and epigenomics can identify genes whose expression and chromatin states are associated with branching, while metabolomics can connect these regulatory changes with branching-related hormones and metabolites; high-throughput phenotyping can quantitatively measure branch number and bud outgrowth, and environmental data can reveal how these relationships vary across growing conditions. Integrating these datasets using GWAS/eQTL, co-expression and regulatory-network, and causal/mediation analyses can prioritize candidate branching regulators, which can subsequently be validated through CRISPR/Cas-mediated editing or other functional-genetic approaches (Tian et al., 2025). Hence, future research must integrate these “omics” approaches to systematically elucidate the molecular “dialogue” between branching-related hormones (e.g., strigolactones & cytokinins) and environmental signals (light, temperature, and nutrition) within the soybean axillary bud micro-environment. This holistic approach will provide a comprehensive theoretical foundation for shaping an “ideal soybean architecture” that balances high yield with stress resilience (**Figure 3C**) (Feng and Khan, 2022; Haidar et al., 2024; Tian et al., 2025; Kundu and Tanti, 2026),.

**Figure 3:**
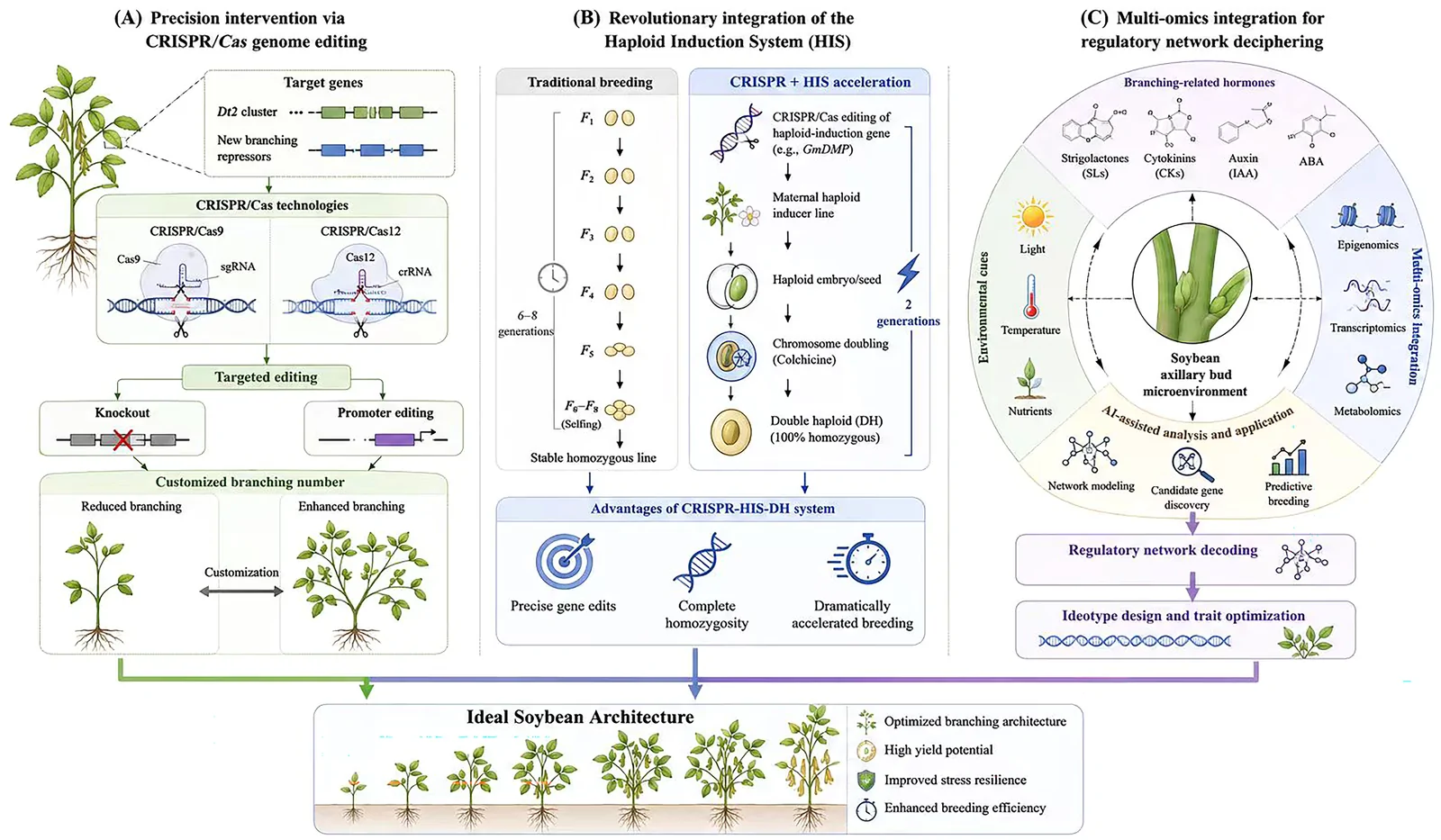
Frontier technologies for shaping ideal soybean architecture. **(A)** Precision genome editing using CRISPR/Cas systems enables targeted knockout or promoter engineering of branching-related genes, including *Dt2* and newly identified branching repressors, thereby facilitating customized optimization of soybean branching architecture. **(B)** The integration of CRISPR-based editing with the Haploid Induction System (HIS) enables the rapid production of homozygous double-haploid (DH) germplasm through *GmDMP*-mediated maternal haploid induction and chromosome doubling, dramatically shortening the breeding cycle. **(C)** Multi-omics approaches integrating epigenomics, transcriptomics, metabolomics, hormonal signaling, and environmental factors provide a systems-level framework for deciphering branching regulatory networks and identifying key determinants of soybean architecture.

Collectively, these emerging technologies offer powerful tools for the rational design of ideal soybean ideotypes with optimized branching patterns, improved yield potential, enhanced stress resilience, and accelerated breeding efficiency. The integration of functional genomics, precision genome editing, and modern breeding strategies is expected to substantially accelerate the development of soybean cultivars with ideal plant architecture adapted to future agricultural demands.

## 6 Conclusion

Plant branching is a core developmental process that determines plant architecture and contributes substantially to final yield. Soybeans have unique developmental and genomic characteristics that may necessitate species-specific regulatory models, despite the fact that conserved regulatory frameworks, such as the *MOC1/OsTB1* network in rice and the *LAS/BRC1* pathway in Arabidopsis, have uncovered important mechanisms governing axillary meristem development and bud outgrowth. The future soybean research should be guided by three priorities: (1) Use genetic and molecular methods to functionally validate branching regulators unique to soybeans and their interactions; (2) elucidate to know whether editing of precise promoter or *cis*-regulatory of dosage-sensitive branching genes can optimize branching-related traits without having undesirable pleiotropic effects; and (3) the edited or naturally existing contrasting branching phenotypes are evaluated across multi-environment trails to unravel stability of branching traits and estimate trade-offs among light interception, branch development, photosynthesis performance, and yield. By combining these strategies, soybean ideotypes with optimized architecture for high-density planting could be developed at a greater pace while retaining yield stability and productivity.
